# Generation of iPSCs as a Pooled Culture Using Magnetic Activated Cell Sorting of Newly Reprogrammed Cells

**DOI:** 10.1371/journal.pone.0134995

**Published:** 2015-08-17

**Authors:** Wenli Yang, Ying Liu, Katherine J. Slovik, Joseph C. Wu, Stephen A. Duncan, Daniel J. Rader, Edward E. Morrisey

**Affiliations:** 1 Institute for Regenerative Medicine, University of Pennsylvania, Philadelphia, PA, United States of America; 2 Department of Medicine, Perelman School of Medicine, University of Pennsylvania, Philadelphia, PA, United States of America; 3 Department of Cell and Developmental Biology, Perelman School of Medicine, University of Pennsylvania, Philadelphia, PA, United States of America; 4 Department of Genetics, Perelman School of Medicine, University of Pennsylvania, Philadelphia, PA, United States of America; 5 Cardiovascular Institute, Perelman School of Medicine, University of Pennsylvania, Philadelphia, PA, United States of America; 6 Division of Cardiology, Department of Medicine; Institute for Stem Cell Biology and Regenerative Medicine, Stanford Cardiovascular Institute, Stanford University School of Medicine, Stanford, CA, United States of America; 7 Program in Regenerative Medicine and Stem Cell Biology, Department of Cell Biology, Neurobiology, and Anatomy, Medical College of Wisconsin, Milwaukee, WI United States of America; Temple University School of Medicine, UNITED STATES

## Abstract

Although significant advancement has been made in the induced pluripotent stem cell (iPSC) field, current methods for iPSC derivation are labor intensive and costly. These methods involve manual selection, expansion, and characterization of multiple clones for each reprogrammed cell sample and therefore significantly hampers the feasibility of studies where a large number of iPSCs need to be derived. To develop higher throughput iPSC reprogramming methods, we generated iPSCs as a pooled culture using rigorous cell surface pluripotent marker selection with TRA-1-60 or SSEA4 antibodies followed by Magnetic Activated Cell Sorting (MACS). We observed that pool-selected cells are similar or identical to clonally derived iPSC lines from the same donor by all criteria examined, including stable expression of endogenous pluripotency genes, normal karyotype, loss of exogenous reprogramming factors, and *in vitro* spontaneous and lineage directed differentiation potential. This strategy can be generalized for iPSC generation using both integrating and non-integrating reprogramming methods. Our studies provide an attractive alternative to clonal derivation of iPSCs using rigorously selected cell pools and is amenable to automation.

## Introduction

The epigenetic reprogramming of somatic cells to a pluripotent state using defined factors was a major advance in stem cell research. Yamanaka and colleagues [1] first reported in 2006 the generation of induced pluripotent stem cells (iPSCs) from fibroblasts by exogenous expression of four transcription factors. Since then, there has been an explosion of research on iPSC technology [2], and it has emerged as a key research tool for studying human disease mechanisms and holds great promise for clinical applications of regenerative medicine [3, 4].

Although significant advancement has been made in the iPSC field, current methods for generating iPSCs are labor intensive, time-consuming and rely heavily on the experience of the researcher for selection of reprogrammed colonies. Most methods involve manually picking multiple colonies and passaging the colonies several times before further expanding them using enzymatic passaging methods to establish iPSC lines. Several studies have shown that there is substantial clone-to-clone variability in the efficiency of differentiation of embryonic stem cells (ESCs) and iPSCs into various cell lineages and the functional properties of the differentiated cells [5–8]. These intrinsic clonal differences necessitate the establishment and characterization of multiple clones from each donor for subsequent studies in order to reach statistically significant and meaningful experimental outcomes. Thus, this labor intensive and time consuming approach of reprogramming substantially hampers the feasibility of large-scale studies where iPSC lines need to be derived from large patient populations. In order to overcome these limitations, more efficient and better-standardized methods for iPSC generation are required.

Strategies have been described that utilize Fluorescence Activated Cell Sorting (FACS) to sort out individual reprogrammed cells that have a defined pluripotency signature [9, 10]. While this “non-manual” method of iPSC colony isolation is highly standardized and can be automated, these studies mainly focused on subsequent expansion and characterization of multiple clones, which are still labor intensive and time consuming, instead of culturing the FACS selected pluripotent cell population as a pooled culture. Willmann et al. recently described a method [11] of simple repeated passaging of initial iPSC colonies in bulk culture without any selection for establishing iPSC lines. While this study points to the use of pooled iPSCs as a faster and more convenient alternative for iPSC generation, the lack of any selection to obtain the cell pools is a potential cause for concern and may result in a heterogeneous cell mixture. We extend the study by Willmann et al. and show here that using Magnetic Activated Cell Sorting (MACS), rigorous selection of TRA-1-60 or SSEA4 positive cells as a pooled culture can be used to establish high quality iPSCs. When compared to clonally derived iPSC lines from the same donor, iPSC pools and clones are highly similar in pluripotency gene expression and spontaneous and lineage directed differential potential. Cell pools also maintain stable expression of pluripotency marker expression over long-term culture and are karyotypically normal. This method provides a fast and efficient alternative to traditional iPSC generation and facilitates automation, which is amenable to rapid generation of iPSCs from large patient populations.

## Results

### Generation of iPSC pools by MACS of TRA-1-60 and SSEA4 positive cells

To generate a homogenous-pooled culture of iPSCs, we sought to use MACS to sequentially select cells that express high levels of cell surface pluripotency markers for the following reasons. MACS is simple and fast to perform and can be easily carried out in the cell culture hood [12]. In addition, MACS allows processing of multiple samples simultaneously, thus increasing throughput. In general, MACS places less shear stress on cells than FACS-based methods, leading to higher cell survival and viability [12]. Since cell surface antigens TRA-1-60 and SSEA4 have been shown to be markers of *bona fide* pluripotent cells [9, 13], we used magnetically conjugated antibodies against either of these two markers to enrich for iPSCs in batch format from a pool of putative newly formed iPSC colonies.

**Fig 1:**
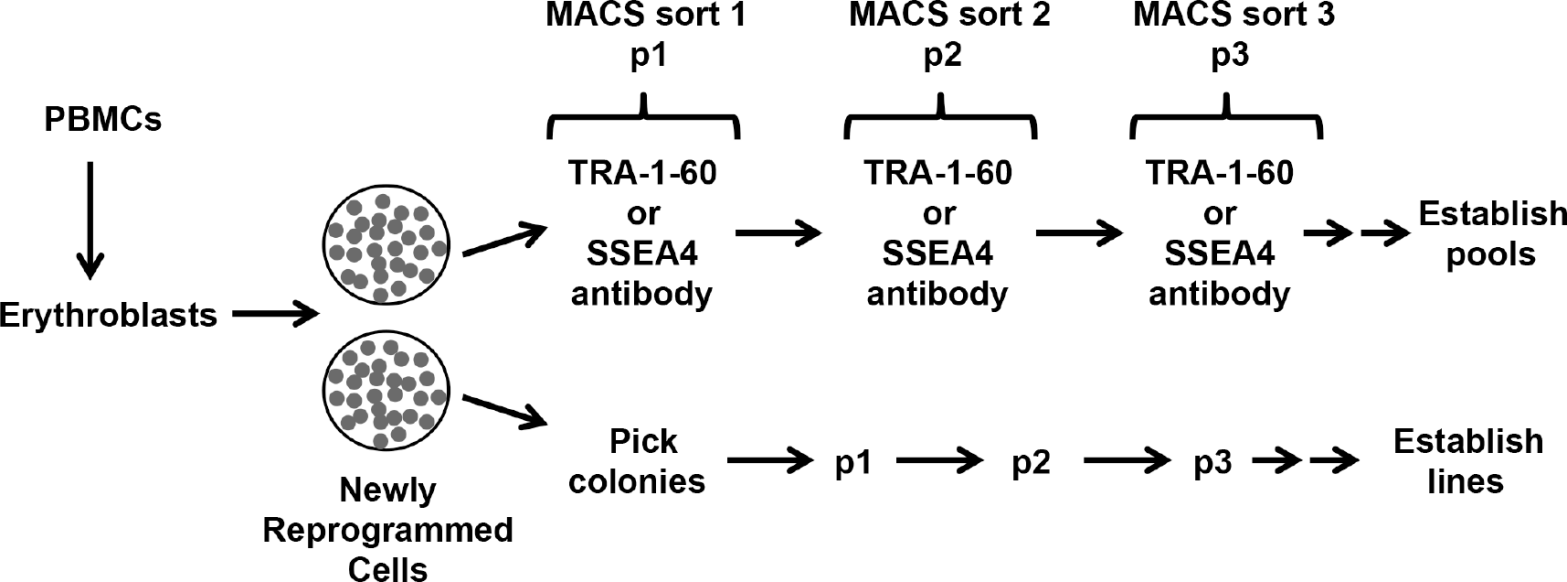
iPSC pool purification scheme using Magnetic Activated Cell Sorting (MACS) of TRA-1-60 or SSEA4 expressing cells. Colonies from a duplicate reprogramming plate were also manually picked and expanded in parallel to establish clonally derived iPSC lines.

Fig 1 shows a flow chart of our reprogramming and iPSC pool purification scheme. We routinely use peripheral blood mononuclear cells (PBMCs) isolated from fresh blood for iPSC generation [14]. PBMCs are cultured first for 7–10 days to allow expansion of erythroblasts. When the cultures are greater than 90% enriched for erythroblasts, they are transduced with Sendai viral reprogramming vectors [15]. For our studies, we reprogrammed cells from the same donors with Sendai virus or hSTEMCCA polycistronic lentiviral reprogramming vector [16, 17] in parallel in order to test MACS purification of iPSCs derived by two different reprogramming methods. Three weeks after initiation of reprogramming, multiple colonies were manually picked from one of two duplicate reprogramming plates and individual iPSC lines were established. To select pluripotent stem cells from a pool of newly reprogrammed colonies, cells from the duplicate plate were dissociated into single cell suspensions, separated into two groups and labeled with either anti-TRA-1-60 or anti-SSEA4 antibodies conjugated to magnetic microbeads. After magnetic sorting, TRA-1-60 or SSEA4 positive cells were then cultured for one passage before an additional round of sorting was performed either with the same antibody used for the first round or the alternate antibody. Each cell pool complexity represented at least 100 distinct iPSC- like colonies by morphology before the first round of sorting. For iPSCs generated using Sendai viral vectors, a third round of sorting was employed to further enrich for pluripotent stem cell population since this method produced a more heterogeneous reprogrammed population due to these vectors encoding individual reprogramming factors.

**Fig 2:**
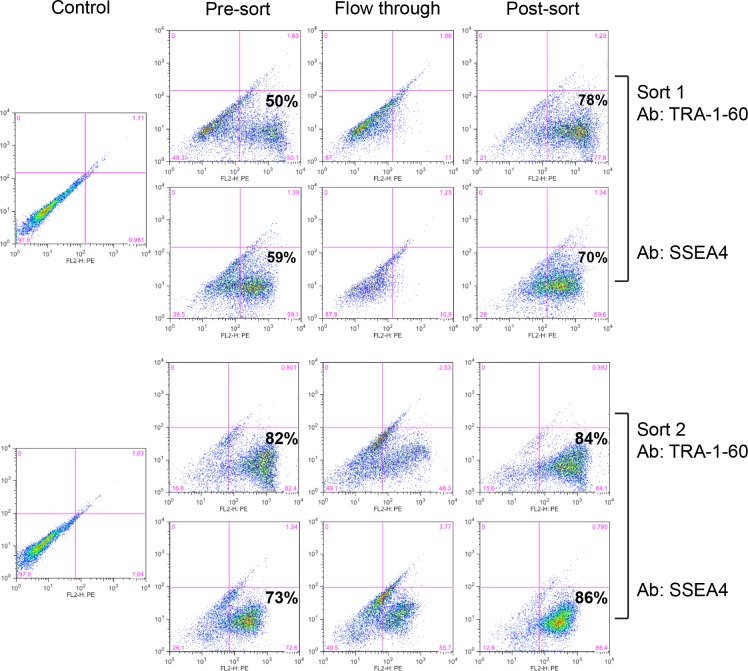
Generation of iPSC pool by MACS from hSTEMCCA reprogrammed cells. Cell populations from before (Pre-sort) and immediately after (Post-sort) MACS, and from the flow through fraction, were analyzed by FACS for enrichment of TRA-1-60 or SSEA4 positive stem cells. Two rounds of MACS were performed. Sort 1 was performed with TRA-1-60 or SSEA-4 antibodies in parallel and sort 2 was performed with either TRA-1-60 or SSEA4 antibody on cells previously sorted with TRA-1-60 antibody.

We analyzed samples from the pre-sort, flow-through and post-sort cell populations by FACS to quantify enrichment of TRA-1-60 or SSEA4 positive cells in the hSTEMCCA reprogrammed cells (Fig 2). The first sort with TRA-1-60 antibody resulted in enrichment from 50% to 78% of TRA-1-60 positive cells while the cells in the flow-through were mostly negative for TRA-1-60 expression. Similarly, the first sort with SSEA4 antibody resulted in 59% to 70% enrichment of SSEA4 expressing cells. Although the initial MACS using either antibody led to a significant enrichment of reprogrammed iPSCs, sorting with the TRA-1-60 antibody appeared to produce slightly better results. However, an additional round of sorting using the TRA-1-60 antibody sorted cells with either TRA-1-60 antibody again or SSEA4 antibody did not further enrich for TRA-1-60 or SSEA4 positive cells significantly (82% to 84% for TRA-1-60 sort and 73% to 86% for SSEA4 sort). These results show that one round of sorting of iPSC cells generated using hSTEMCCA may be sufficient to produce a homogenous population of iPSCs.

**Fig 3:**
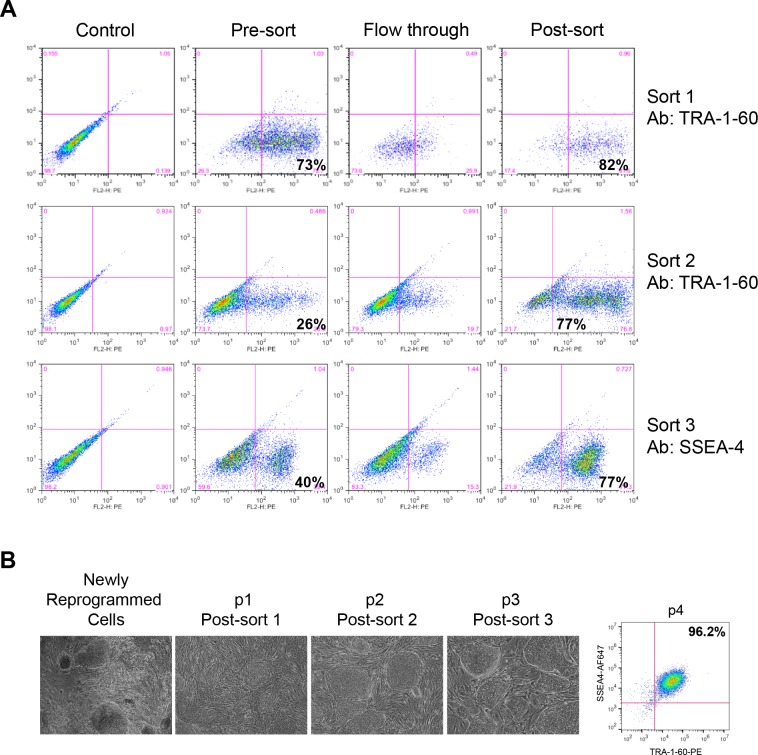
Generation of iPSC pool by MACS from Sendai virus reprogrammed cells. (**A**) Cell populations from before (Pre-sort) and immediately after (Post-sort) MACS, and from the flow through fraction, were analyzed by FACS for enrichment of TRA-1-60 or SSEA4 positive stem cells. Three rounds of MACS were performed. Sort 1 was performed with TRA-1-60 antibody; TRA-1-60 positive cells were then used for sort 2 with TRA-1-60 antibody again; and the resulting cells were used for sort 3 with SSEA4 antibody. (**B**) Phase contrast pictures of newly reprogrammed colonies before MACS and sorted cells cultured for 4 days after each round of antibody sorting (Post-sort). FACS plot of p4 Sendai virus reprogrammed cell pool after 3 rounds of sorting showing percentage of SSEA4+TRA-1-60+ cells (right panel).

Based on the results obtained with hSTEMCCA-derived cells, we performed the first sort of Sendai virus reprogrammed cells with the TRA-1-60 antibody. The percentage of TRA-1-60 expressing cells in the initial unsorted cells was high (73%) but the expression level was variable, and the first round of sorting did not result in significant enrichment (73% to 82%, Fig 3A). However, after one passage, the percentage of TRA-1-60 positive cells decreased to 26% (Fig 3A), presumably due to the overgrowth of non-reprogrammed or partially reprogrammed cells. A second round of sorting with TRA-1-60 antibody similarly led to enrichment of 77% positive cells. However, after one more passage, the cell population only stained 40% positive for SSEA4. We therefore performed a third round of sorting with the SSEA4 antibody, which led to enrichment from 40% to 77% SSEA4 positive cells with tight expression of SSEA4. Fig 3B shows the morphology of Sendai viral vector derived cells after each sort following culture for 4 days. While post-sort 1 and post-sort 2 cultures were still heterogeneous in morphology, the post-sort 3 cultures uniformly consisted of colonies with iPSC morphology (Fig 3B). We performed FACS analysis of the sorted cells at p4 and found that more than 96% of the cells are SSEA4+TRA-1-60+, showing strong evidence that three rounds of MACS is enough for iPSC pool generation. We therefore passaged the post-sort 3 cells further for establishment of iPSC pools.

We were also able to establish an iPSC pool using the same scheme by re-plating a culture of newly emerged iPSC colonies, previously frozen at 3 weeks of reprogramming. First, cells were thawed and grown to confluence; three rounds of MACS purification were then performed on the cells followed by FACS analysis. Although the starting numbers of TRA-1-60+SSEA+ cells are low (2.61%) due to the growth advantage of partially reprogrammed cells from the thawed culture, three rounds of sorting was enough to establish a homogeneous culture of 90% TRA-1-60+SSEA+ cells (S1A Fig). It is well established that complete reprogramming of somatic cells back to the pluripotent state requires extensive molecular changes which take multiple passages of newly formed iPSCs to complete. Since we used CD71+CD36+ erythroblast cultures expanded from PBMCs for reprogramming [14], we therefore analyzed the expression of CD71 in post-sort 3 TRA-1-60+SSEA+ cells and found that about 31% of the double positive cells expressed low levels of CD71 (S1B Fig). This suggests that these cells are transitioning from erythroblasts to iPSCs at this early stage of passage 3.

### Pool derived iPSCs maintain pluripotency and genomic integrity

**Fig 4:**
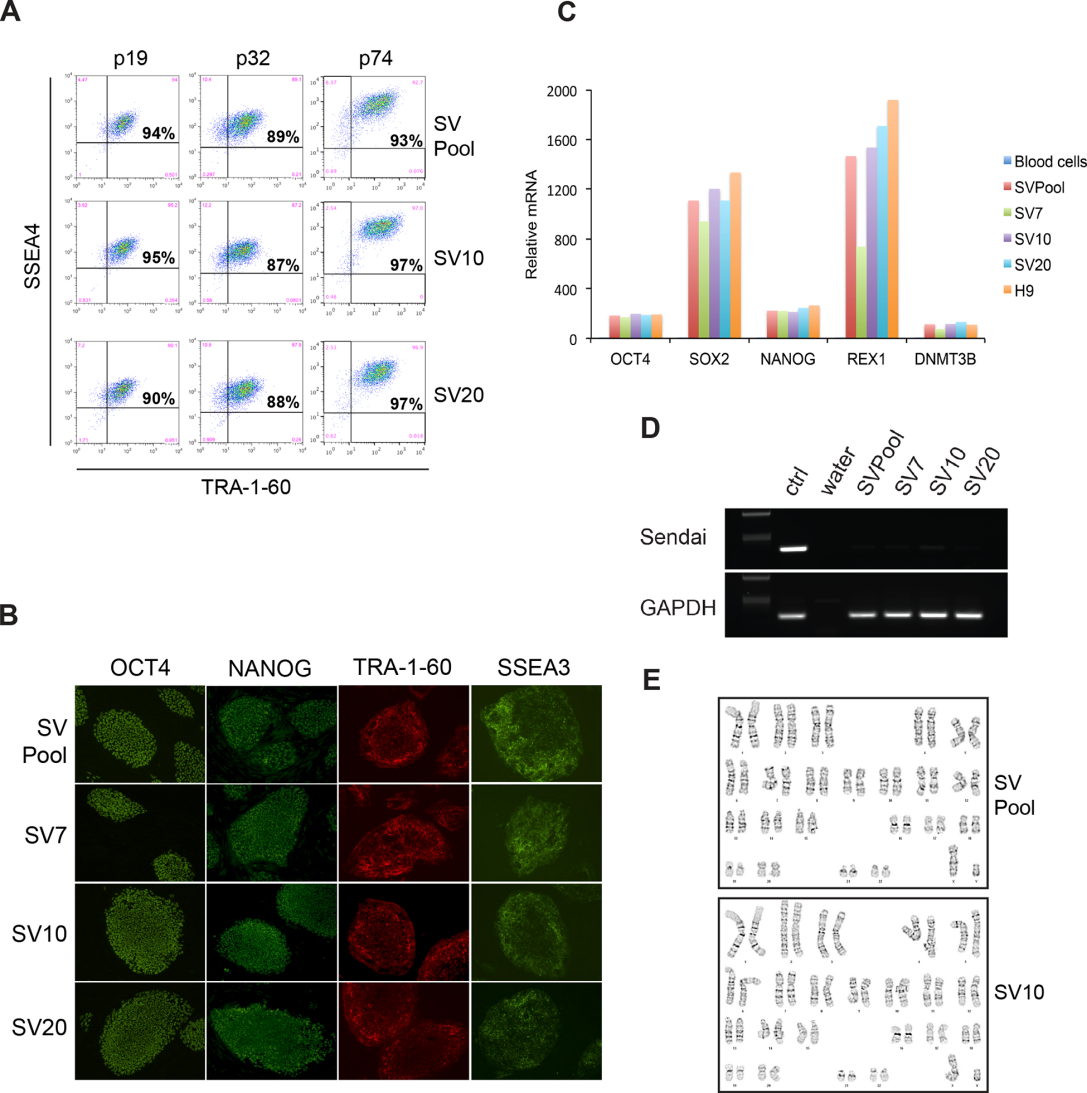
Characterization of MACS purified iPSC pool and clones. (**A**) FACS analysis showing pool selected iPSCs (SVPool) stably express pluripotency markers TRA-1-60 and SSEA4 through long-term passaging up to p74, similar to three clonally derived iPSC lines (SV7, SV10, SV20) from the same donor. (**B**) Immunocytochemistry and (**C**) qRT-PCR analyses of mRNA levels from iPSCs showing SVPool and iPSC clones (p15–20) express similar levels of a panel of pluripotency markers tested. (**D**) SVPool and iPSC clones both show Sendai viral reprogramming vector clearance at p15–20 by RT-PCR analysis. (**E**) G-banded karyotyping was performed on SVPool and clones at p28–33 and all cell lines were found to be karyotypically normal. The data presented are chromosomal banding patterns of SVPool and one of the clones (SV10) for comparison.

We performed extensive characterization of the Sendai virus reprogrammed iPSC pool and compared to individual iPSC clones derived from the same blood sample. Three different individual iPSC clones (SV7, SV10, SV20) were compared to an iPSC pool (SVPool). The SVPool and individual clones all maintained a high percentage of TRA-1-60 and SSEA4 double positive cells up to at least 74 cell passages, suggesting that iPSC pools are phenotypically stable over time (Fig 4A). Immunostaining and qRT-PCR of a panel of pluripotency markers show that the SVPool expressed similar levels of these markers compared to individual clones (Fig 4B and 4C). The H9 hESC line [18] was used as a positive control for gene expression analysis in these assays. Both the SVPool and iPSC clones showed a complete loss of Sendai viral vectors as expected at passages 10–12 (Fig 4D). SVPool and the iPSC clones are also karyotypically normal as determined by G-banding (Fig 4E). Taken together, these results demonstrate that iPSC pools generated using MACS are similar to clonally derived iPSCs. Pool derived iPSCs stably express pluripotency genes after long term passage, exhibit normal karyotype and uniformly lose exogenous reprogramming factor expression.

### Pool derived iPSCs have similar differentiation potential as their clonally derived counterparts

**Fig 5:**
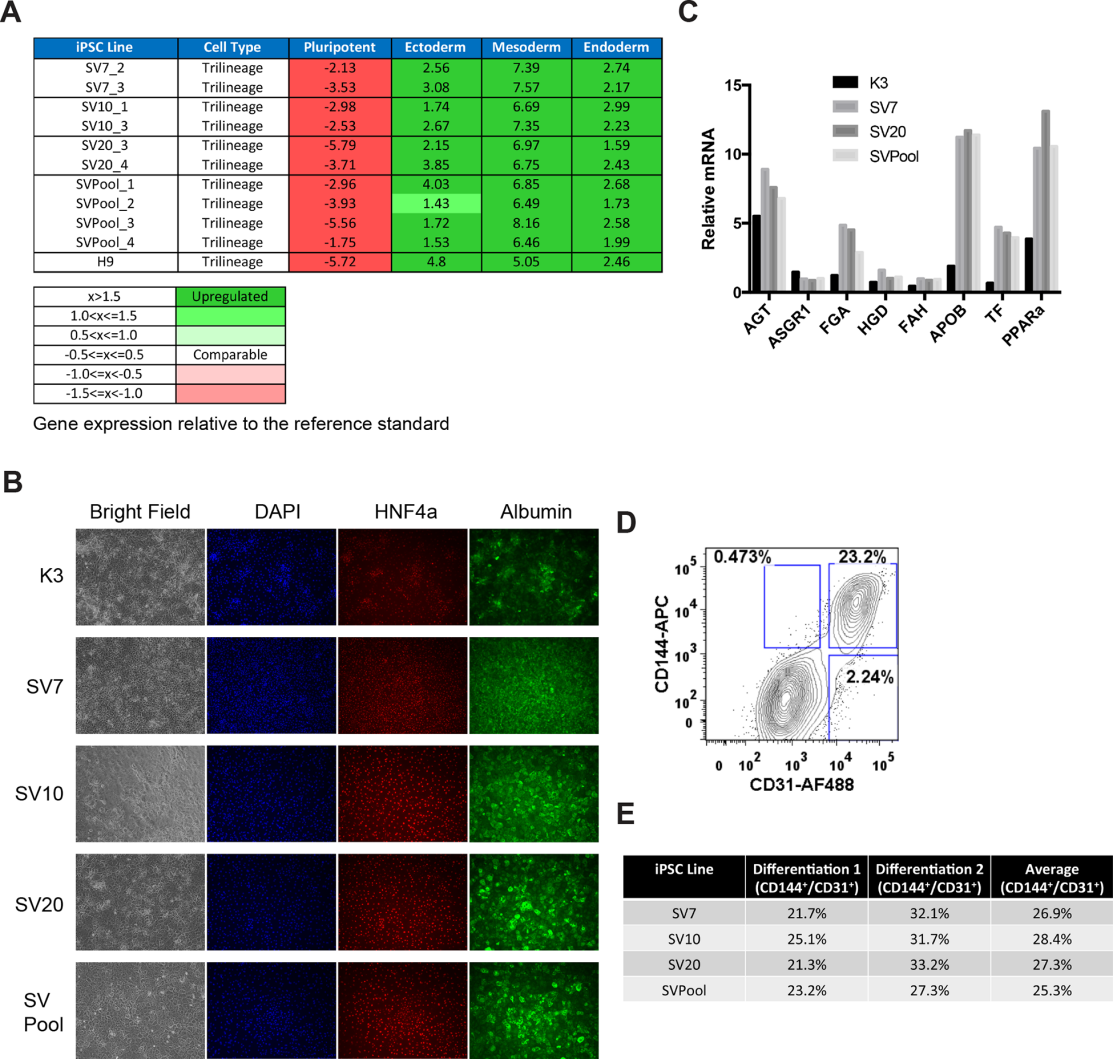
Comparison of *in vitro* differentiation potential of iPSC pool and clones. (**A**) iPSC pool (SVPool) and clones (SV7, SV10, SV20) were induced to differentiate by embryoid body (EB) formation assay for 14 days. Multiple differentiations were performed for each cell line between p20–30. Total RNA isolated from the EBs was used for hPSC Scorecard analysis for tri-lineage differentiation potential. Scores for expression of pluripotency, ectoderm, mesoderm and endoderm lineage genes were calculated relative to a set of reference hESC and iPSC lines. Scores for the hESC line H9 was used for comparison. SVPool and all iPSC clones show similar differentiation potential. (**B**) SVPool and clones were induced to differentiate into hepatocyte-like cells. The iPSC line K3 was used as a positive control. Immunocytochemistry of day 21 differentiated hepatocytes show comparable expression of hepatocyte markers HNF4α and Albumin in all cell lines. (**C**) Taqman qRT-PCR analysis of mature hepatocyte markers show similar gene expression levels among hepatocytes derived from SVPool and iPSC clones. mRNA levels were expressed as fold changes relative to those of human primary hepatocytes. The iPSCs were induced for endothelial cell differentiation. At day 14 of differentiation, cells were sorted for CD31^+^CD144^+^ cells using FACS. A representative FACS plot of the percentage of CD31^+^CD144^+^ cells in the differentiation culture (**D**) and similar differentiation efficiencies (percentages) amongst SVPool and clones from two independent experiments (**E**) are shown.

To determine whether pool derived iPSCs have a similar ability to differentiate into multiple cell lineages as their clonally derived counterparts, we performed *in vitro* spontaneous differentiation using the embryoid body (EB) formation assay as well as lineage directed differentiation. EBs were generated in suspension culture for 14 days. RNA from the EBs was then isolated and subjected to the hPSC ScoreCard assay, originally developed by the Meissner laboratory [19] to assess pluripotency and tri-lineage differentiation potential. We found that SVPool and all the iPSC clones exhibit similar potential for generating cell lineages from all three germ layers (Fig 5A). We also generated EBs from the H9 hESC line as a positive control and found that our iPSC clones and SVPool scored similarly to the H9 derived EBs in this assay (Fig 5A).

Next, the iPSC pool and clones were compared in a lineage specific differentiation assay. Directed differentiation into hepatocytes, an endoderm derived cell type, was performed using a protocol we previously described [20, 21]. Using this protocol, the SVPool cells converted into Albumin and HNF4α expressing hepatocyte-like cells as efficiently as the individual iPSC clones (Fig 5B). This differentiation potential was similar at both early (p20s, data not shown) and later (p71–84, Fig 5B) passages. Moreover, differentiation of the SVPool and clones was as efficient as or more efficient than the previously described control iPSC line K3 [22]. qRT-PCR analysis of an expanded panel of mature hepatocyte-specific marker genes revealed a similar level of expression of these genes in the SVPool and individual clones (Fig 5C, S2 Fig). These results show that SVPool and clones exhibit robust and essentially equivalent differentiation potential into endoderm derived hepatocyte-like cells.

The SVPool and individual clones were also differentiated into endothelial cells, a mesoderm derived cell lineage, using a protocol adapted from a murine iPSC to endothelial cell differentiation protocol that we previously published [23]. At the end of the 14-day procedure, FACS using two endothelial cell markers CD144 and CD31 was performed to quantify differentiation efficiency. We found that SVPool cells gave similar percentages of CD144^+^/CD31^+^ cells as the clonally derived iPSCs (Fig 5D and 5E). These endothelial cell differentiation efficiencies are typical of what we routinely obtain for human hESC and iPSCs and what have been previously reported for murine iPSCs [23]. Taken together, these results suggest that pool derived iPSCs have a similar multi-lineage differentiation potential as clonally derived iPSCs.

## Discussion

The classic method of iPSC derivation requires labor-intensive and time-consuming manual isolation, expansion, and characterization of multiple colonies. Thus, better methods need to be developed to facilitate more efficient generation of iPSCs for large-scale studies. Here we report a method of generating iPSCs by magnetic activated cell sorting using TRA-1-60 or SSEA4 antibodies of a reprogrammed cell pool. This method is suitable for selecting *bona fide* iPSCs generated using integrating and non-integrating methods. Comparison of pool-selected cells to isogenic clonally derived lines reveals that pools are highly similar to clones in all characterization criteria assessed. In addition, all of the clones are highly similar to one another using the characterization criteria examined. Our studies provide an attractive alternative to clonal derivation of iPSCs using rigorously selected cell pools. Of note, a recent report also described a similar approach to successfully generate iPSCs as cell pools [24].

We also found that early passage pool selected iPSCs retain expression of CD71, a marker of erythroblast, the somatic cell of origin in these studies. This raises the possibility that an early round of negative selection using CD71 antibody may increase purity of initially sorted pluripotent cell populations and reduce the need for multiple rounds of sorting. A manuscript describing the use of CD71 and CD13 as negative selection markers for establishment of iPSCs from blood derived sources was recently published [25].

Recent studies showed significant variability in differentiation potential among iPSC clones derived from the same donor [5–8]. Moreover, significant variation even at the single reprogrammed cell level has been reported [26]. These clonal differences may arise from DNA sequence variations in the original non-clonal parental cell populations, mutations acquired during the reprogramming process or subsequent expansion and culture, or variations in residual epigenetic signature of the parental cells [8, 27]. These findings have suggested that it is necessary to examine multiple iPSC clones in most phenotyping assays. However, our data using the non-integrating Sendai viral reprogramming vectors suggest that individual clones may not be necessary. The use of pooled iPSCs may substitute for multiple clones since iPSC clones derived from the same individual are remarkably similar to one another and to pool derived iPSCs in their potential to differentiate into multiple lineages. Some of this consistency may be due to the use of non-integrating Sendai virus as a reprogramming method. A previous report using non-integrating episomal vectors also revealed very little clone-to-clone variability in iPSC reprogramming when derived from the same donor [11]. Despite the advantages of using pool derived iPSCs, in studies where phenotypic differences between iPSCs of different genotypes may be small, such subtle changes could be lost in a “pool” of reprogrammed iPSCs. In such cases, the use of multiple clones will be necessary to reach meaningful and statistically significant conclusions. However, increasing the number of biological replicates of iPSC cell pools derived from multiple donors of the same genotype could also alleviate such limitations, especially given the ability for pool selection to be automated.

In summary, we have developed a method of generating iPSCs as reprogrammed cell pools through MACS of TRA-1-60 or SSEA4 positive candidate iPSCs. This method is more efficient, less time consuming and better standardized than clonal selection and may facilitate rapid generation of iPSCs from a large number of patient samples.

## Materials and Methods

### Ethics statement

iPSC generation from human subjects was approved by the University of Pennsylvania Human Subjects Research Institutional Review Board. Written informed consent was obtained from all human cell donors. This study was conducted in accordance with the Declaration of Helsinki.

### iPSC derivation and culture

iPSC derivation from PBMCs using Sendai viral vectors [14] and hSTEMCCA [17] were described in detail previously. All iPSCs were cultured in humidified chambers at 37°C/20% CO_2_/5% O_2_. Unless otherwise stated, hESCs and iPSCs were cultured on irradiated mouse embryonic fibroblast (MEF) feeder cells (Global Stem, Gaithersburg, MD) in hESC medium consisting of DMEM/F12 supplemented with 20% knockout serum replacement (KOSR), 2 mM L-glutamine, MEM non-essential amino acids (NEAA), 1% penicillin/streptomycin (P/S) and 10 ng/mL basic fibroblast growth factor (bFGF). All ingredients for hESC medium were obtained from Life Technologies.

### Establishment of iPSCs by MACS

iPSCs were dissociated into single cells with Accutase Enzyme Cell Detachment Medium (Innovative Cell Technologies). Cell suspensions were then passed through a pre-separation filter (Miltenyi Biotec) to remove cell aggregates and cell counts were obtained. Up to 2x10^6^ cells were resuspended in 100 μL of hESC medium supplemented with 5 μM ROCK inhibitor Y-27632 (Enzo Life Sciences) and incubated for 10 min at 4°C with 10 μL of anti-TRA-1-60-PE or anti-SSEA4-PE antibody (Miltenyi Biotec). Typically 2–3 wells of a 6-well plate containing the original reprogrammed colonies were used for the first round of sorting. After antibody labeling, the cells were washed with 2 mL of hESC medium and incubated for 15 min at 4°C with 20 μL of anti-PE magnetic microbeads (Miltenyi Biotec) in 80 μL of fresh hESC medium. After washing, cells were suspended in 0.5 mL fresh hESC medium supplemented with 5 μM Y-27632 and loaded onto LS separation columns (Miltenyi Biotec) and antibody labeled cells were retained under a magnetic field. After extensive washing of the column with hESC medium, antibody labeled cells were eluted in fresh hESC medium and directly plated for culture.

### FACS analysis

Assessment of cell surface markers expression by FACS was performed using a FACSCalibur or an Accuri C6 flow cytometer (BD Biosciences). Antibodies used are anti-SSEA4-PE (Miltenyi Biotec), anti-TRA-1-60-PE (Miltenyi Biotec), anti-SSEA4-AF647 (Biolegend), anti-TRA-1-60-FITC (BD Biosciences) and anti-human CD71-PE (Biolegend). FACS data were analyzed using FlowJo software (Tree Star).

### Real time qRT-PCR and RT-PCR

Total RNA was isolated using an RNAeasy kit (Qiagen) according to manufacturer’s instructions and cDNA synthesis was primed via random priming. Real time qRT-PCR for expression of pluripotency genes was performed using SYBR green reagents (Applied Biosystems) and specific primers for endogenous OCT4, endogenous SOX2, NANOG, REX1 and DNMT3B genes (S1 Table). Taqman based qRT-PCR (Applied Biosystems) was used for gene expression analysis of hepatocyte markers using PrimeTime assay primers (IDT; S2 Table). RNA isolated from primary human hepatocytes obtained from Life Technologies was used for comparison. RT–PCR for confirmation of loss of Sendai viral reprogramming vectors was performed with primer sets against the Sendai viral genome (forward primer: 5’-GGATCACTAGGTGATATCGAGC-3’ and reverse primer: 5’-ACCAGACAAGAGTTTAAGAGATATGTATC-3’) and GAPDH as a normalization control (forward primer: 5’-GTGGACCTGACCTGCCGTCT-3’ and reverse primer: 5’-GGAGGAGTGGGTGTCGCTGT-3’). PCR products were analyzed using 2% agarose gel electrophoresis.

### Immunofluorescence staining

iPSCs at passages 15–20 were grown to confluence in 48-well tissue culture plates on mouse feeders. Cells were fixed in 4% paraformaldehyde and permeabilized with 0.2% Triton X-100. Indirect immunofluorescence staining was then performed with antibodies against OCT3/4 (Abcam), SOX2 (R&D systems), SSEA3 (Millipore) and NANOG (Abcam), followed by Alexa fluorochrome-conjugated secondary antibodies (Life Technologies). Results were observed using Nikon Eclipse Ti-U inverted microscope with DS-Qi1 monochrome digital camera. The procedure used to stain for HNF4a and Albumin expression has been described in detail previously [28].

### Karyotyping analysis

At cell passages 29–35, iPSCs cultured in mTeSR1 (Stemcell Technologies) medium on Matrigel (BD Biosciences)-coated T25 flasks were sent to WiCell (Madison, WI) as live cultures for cytogenetic analysis using G-banded karyotyping. An average of 20 cells per cell line were analyzed for chromosome integrity.

### Embryoid body formation and hPSC ScoreCard analysis

Embryoid body formation was performed essentially as described [19]. iPSCs were grown on MEF feeders to confluence and harvested as large clusters using 1mg/mL dispase (Stemcell Technologies). Cell clusters were then cultured in suspension in EB20 medium (DMEM/F12 supplemented with 20% fetal bovine serum (FBS; Hyclone), NEAA, L-glutamine and P/S) in 6-well ultra low attachment plates (Corning) to induce differentiation. Fresh medium was replaced every three days. After 14 days of culture, EBs were collected and total RNA was isolated and subjected to hPSC ScoreCard (Life Technologies) analysis according to manufacturer’s instructions. ScoreCard is a Taqman based qRT-PCR assay that assesses pluripotency and trilineage differentiation propensity by comparing the expression of a panel of 74 genes of the test cells to that of a reference set of standard iPSC and hESC lines. Briefly, 1 μg of total RNA from each sample was used for cDNA synthesis and subsequent Taqman qRT-PCR analysis in 384- well format plates. Data were analyzed using a cloud-based software and results were expressed as a score relative to the reference set.

### Directed hepatocyte differentiation

iPSC derived Hepatocyte differentiation was performed as described [21]. Briefly, iPSCs were cultured on an E-Cad-Fc substrate (Stemcell Technologies) before transferred to Matrigel-coated dishes and cultured overnight in mTeSR1 medium to near confluence. To induce differentiation, cells were exposed to a cocktail of 100 ng/mL Activin A (R&D System, 20 ng/mL bFGF (Life Technologies) and 10 ng/mL BMP4 (R&D Systems) in RPMI medium (Life Technologies) with B-27 supplement (minus insulin) (Life Technologies) for 3 days. This was then followed by culturing in RPMI/B27 (minus insulin) supplemented with 100 ng/mL Activin A alone for an additional 2 days to induce definitive endoderm formation. The media was then changed to RPMI/B-27 (with insulin) (Life Technologies) plus 10ng/mL bFGF and 20 ng/mL BMP4 for 5 days to induce hepatic progenitor cell formation. The cells were then cultured in RPMI/B-27 (with insulin) plus 20 ng/mL HGF (Peprotech) for 5 days to induce for immature hepatocytes. Finally, mature hepatocytes were formed and maintained in HCM media (minus EGF) (Lonza) supplemented with 20 ng/mL Oncostatin-M (R&D Systems) for 5 days. At this point, hepatocytes were fixed and subjected to immunostaining or harvested for RNA isolation and subsequent real time qRT-PCR analysis.

### Directed endothelial cell differentiation

iPSC derived endothelial cell differentiation was performed essentially as described [23]. To induce endothelial cell differentiation, approximately 1 x 10^5^ human iPSCs were seeded in each well of Matrigel-coated 6-well plates and cultured in differentiation medium containing RPMI and B-27 supplement (minus insulin) with 5 μM CHIR-99021 (a glycogen synthase kinase [GSK]-3 inhibitor; Selleck Chemicals, Houston, TX) for 2 days, followed by RPMI/B27 (minus insulin) with 2 μM CHIR-99021 for 2 additional days. The medium was then changed to RPMI/B-27 (minus insulin) with 25 ng/mL vascular endothelial growth factor (VEGF; R&D Systems) and 10 ng/mL bFGF (R&D Systems) for 3 days. Finally, for the subsequent 7 days the medium was changed to RPMI/B27(with insulin) with 25 ng/mL VEGF and 10 ng/mL bFGF. At day 14, human iPSC-ECs were sorted for CD31^+^/CD144^+^ cells using FACS. Antibodies against CD31 and CD144 were obtained from Biolegend.

## Supporting Information

S1 Fig Establishment of an iPSC pool from previously frozen primary iPSC colonies by MACS. (**A**) A pool of newly emerged iPSC colonies reprogrammed from a patient blood sample (M11) were cryopreserved at 3 weeks after initiation of reprogramming. The cells were thawed and plated on MEF feeders and grown to confluency. The cells were then subjected to MACS purification scheme as described in Fig 1 and an iPSC pool was established after three rounds of cell sorting. FACS plots showing SSEA4+TRA-1-60+ cell populations of plated cultures 4–5 days after each sort are shown. Enrichment from 2.61% to 90.0% SSEA4+TRA-1-60+ cells is achieved after three sorts. (**B**) Post-sort 3 (p3) cells were also stained with anti-human CD71 antibody. The percentage of CD71- and CD71+ populations in the SSEA4+TRA1-60+ cell population is shown.
(TIF)

S2 Fig Comparison of hepatocyte differentiation efficiency of iPSC pool and clones. Taqman qRT-PCR analysis of a panel of hepatocyte marker genes that reflect various aspects of the mature cell phenotype show similar gene expression levels among clones and pool. mRNA levels are expressed as fold changes relative to the levels in freshly isolated human primary hepatocytes. A previously published iPSC line K3 (from a different donor) was used as a positive control of differentiation.
(TIF)

S1 Table Real time qRT-PCR primers used for analysis of pluripotency gene expression. (DOCX)

S2 Table PrimeTime assays for Taqman based qRT-PCR analysis of hepatocyte markers. Catalogue number for each assay is listed. Assays were purchased from IDT.
(DOCX)
