# Supplementary figures and images for: Generation of iPSCs as a Pooled Culture Using Magnetic Activated Cell Sorting of Newly Reprogrammed Cells
Source: PLoS One. 2015 Aug 17;10(8):e0134995. doi: 10.1371/journal.pone.0134995 (PMC4539221; doi:10.1371/journal.pone.0134995)

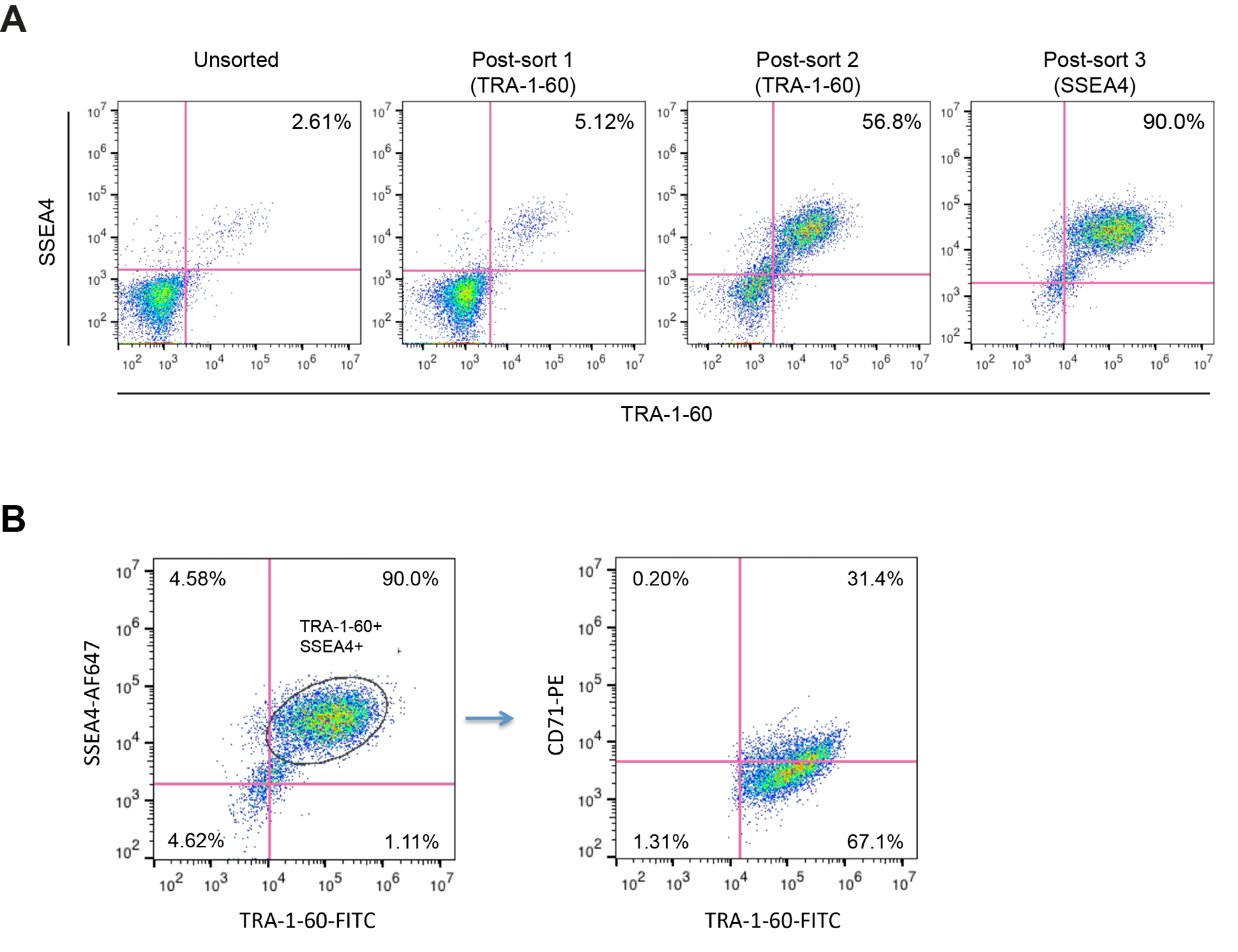

Supplement: S1 Fig — (A) A pool of newly emerged iPSC colonies reprogrammed from a patient blood sample (M11) were cryopreserved at 3 weeks after initiation of reprogramming. The cells were thawed and plated on MEF feeders and grown to confluency. The cells were then subjected to MACS purification scheme as described in Fig 1 and an iPSC pool was established after three rounds of cell sorting. FACS plots showing SSEA4+TRA-1-60+ cell populations of plated cultures 4–5 days after each sort are shown. Enrichment from 2.61% to 90.0% SSEA4+TRA-1-60+ cells is achieved after three sorts. (B) Post-sort 3 (p3) cells were also stained with anti-human CD71 antibody. The percentage of CD71- and CD71+ populations in the SSEA4+TRA1-60+ cell population is shown. (TIF) [file pone.0134995.s001.tif]

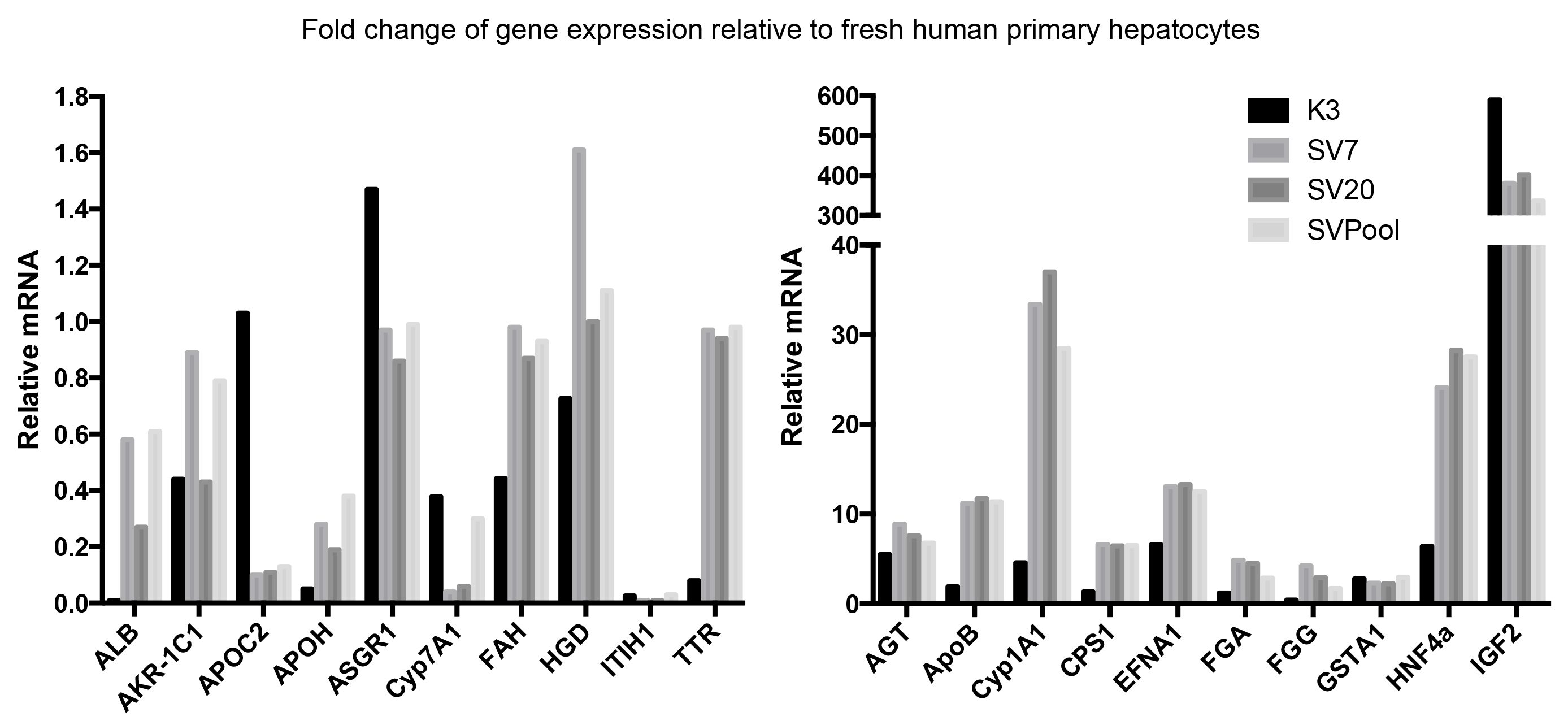

Supplement: S2 Fig — Taqman qRT-PCR analysis of a panel of hepatocyte marker genes that reflect various aspects of the mature cell phenotype show similar gene expression levels among clones and pool. mRNA levels are expressed as fold changes relative to the levels in freshly isolated human primary hepatocytes. A previously published iPSC line K3 (from a different donor) was used as a positive control of differentiation. (TIF) [file pone.0134995.s002.tif]
