# Supplementary material for: Generation of iPSCs as a Pooled Culture Using Magnetic Activated Cell Sorting of Newly Reprogrammed Cells
Source: PLoS One. 2015 Aug 17;10(8):e0134995. doi: 10.1371/journal.pone.0134995 (PMC4539221; doi:10.1371/journal.pone.0134995)
Supplement: S1 Table — (DOCX) [file pone.0134995.s003.docx]

**S1 Table.** **Real time qRT-PCR primers used for analysis of pluripotency gene expression**

| **Gene** | **Sequence** |
| --- | --- |
| endo-OCT4 | 5'-AAC CTG GAG TTT GTG CCA GGG TTT-3' |
|  | 5'-TGA ACT TCA CCT TCC CTC CAA CCA-3' |
| endo-SOX2 | 5'-AGA AGA GGA GAG AGA AAG AAA GGG AGA GA-3' |
|  | 5'-GAG AGA GGC AAA CTG GAA TCA GGA TCA AA-3' |
| NANOG | 5'-CCA ACA TCC TGA ACC TCA GC-3' |
|  | 5'-GCT ATT CTT CGG CCA GTT G-3' |
| REX1 | 5'-GCG TCA TAA GGG GTG AGT TTT-3' |
|  | 5'-AGA ACA TTC AAG GGA GCT TGC-3' |
| DNMT3B | 5'-TAC AGA CGT GTG CAG TTG TAG GCA-3' |
|  | 5'-GTG CAG ACT CCA GCC CTT GTA TTT-3' |
