# Supplementary material for: Generation of iPSCs as a Pooled Culture Using Magnetic Activated Cell Sorting of Newly Reprogrammed Cells
Source: PLoS One. 2015 Aug 17;10(8):e0134995. doi: 10.1371/journal.pone.0134995 (PMC4539221; doi:10.1371/journal.pone.0134995)
Supplement: S2 Table — Catalogue number for each assay is listed. Assays were purchased from IDT. (DOCX) [file pone.0134995.s004.docx]

**S2 Table. PrimeTime assays for Taqman based qRT-PCR analysis of hepatocyte markers.** Catalogue number for each assay is listed. Assays were purchased from IDT.

| **Gene** | **PrimeTime Catalogue Number** |
| --- | --- |
| AGT | N000029.1.pt.AGT |
| ApoB | Hs.PT.47.18897448 |
| ASGR1 | 107819464 |
| FAH | Hs.PT.45.533899 |
| FGA | N021871.1.pt.FGA |
| HGD | Hs.PT.51.2181009 |
| PPARa | N005036.1.pt.PPARA |
| TF | N001063.1.pt.TF |
